# Factors affecting nursing practice of patient physical restraint among nurses

**DOI:** 10.1186/s13690-024-01238-z

**Published:** 2024-01-16

**Authors:** Jihyun Kim, Yaki Yang

**Affiliations:** 1 Department of Nursing, Kunsan College of Nursing, Kunsan, South Korea; 2 Department of Nursing, College of Medicine, Wonkwang University, Iksan, South Korea

**Keywords:** Physical restraint, Nurse, Perception, Knowledge, Attitude, Nursing practice

## Abstract

**Background:**

This study was aimed to identify perception, knowledge, attitude and nursing practice toward use of physical restraints among clinical nurses.

**Methods:**

The research participants were 180 nurses from general hospitals located in Korea. Data were collected using self-report questionnaires regarding perception, attitude, knowledge, and nursing practice on application of physical restraints and analyzed using t-test, ANOVA, Pearson correlation coefficients, and multiple regression.

**Results:**

There were significant negative relationships attitudes towards the use of physical restraints with knowledge (*r* = -.32, *p* < .001). Knowledge showed a positive correlation with nursing practice (*r* = .28, *p* < .001). Factors affecting nursing practice of clinical nurses were identified as knowledge (β = .23), education experiences on physical restraints (Yes) (β = .18), and work unit (ICU) (β = .43). The explanation power of this regression model was 22% and it was statistically significant (*F* = 7.45, *p* < .001).

**Conclusion:**

This study suggests that knowledge, education experiences on physical restraints, and work unit were the strongest predictor on nursing practice toward use of patient physical restraints. Therefore, developing and applying evidence-based educational intervention programs by work unit to reduce the inappropriate use of physical restraints in hospitals are required.

**Table:** 

**Text box 1. Contributions to the literature**
• The Nurses must be able to make appropriate decisions about the application and management of physical restraints in a variety of clinical situations.
• It is significant in that knowledge of physical restraints, educational experience, and work units are the strongest predictors of nursing practice for patients' use of physical restraints.
• It is essential and urgent to prepare guidelines for the application of physical restraints, taking into account variations in work unit and assigned responsibilities.

## Introduction

Physical restraints refer to passive methods or physical devices and equipment used to restrict the movement of the body in order to protect the patient or others for medical purpose [1]. Initially, physical restraints were primarily used in psychiatric settings to control the behavior of mentally ill patients exhibiting aggressive behavior. However, with the development of various anti-psychotic medications, their use in psychiatric settings has gradually decreased. In recent years, they have been applied in general wards for the purpose of fall prevention, and in intensive care units to reduce the risk of falls and prevent the removal of life-supporting devices, aiming to protect patients and control aggressive behavior [2, 3]. In the United States and Canada, the application rate of physical restraints in intensive care units is approximately 7–8 times higher than in general wards, and in Korea intensive care units, it has been reported to be more than 10 times higher than in general wards [4, 5].

However, the application of physical restraints used for patient safety has been consistently reported to have physical complications such as musculoskeletal system deterioration associated with immobility, aspiration pneumonia, nerve damage, skin injuries, and suffocation-related death due to chest compression [6, 7]. It also causes side effects such as fear, depression, confusion, and increased aggression, leading to psychological problems [6, 7]. Recently, there has been increasing attention to the physical, emotional, and psychological side effects of physical restraint use, as well as issues related to patient freedom and human rights. Major countries have actively pursued the reduction and regulation of physical restraint use. In the United States, organizations such as the Joint Commission on the Accreditation of Healthcare Organizations to regulate the misuse of physical restraints [8].

In Korea, it was not until 2007 that the Ministry of Health and Welfare established guidelines for the use of physical restraints in healthcare facilities under the category of "Physical restraint and stabilization management." These guidelines aimed to assess the appropriateness of restraint use and the adequate management of patients after the application of restraints in comprehensive hospitals. Since 2015, items related to physical restraints have been included in the evaluation criteria for healthcare facilities, which assess the appropriateness of their use and the management of restrained patients [9]. However, regulations and guidelines regarding the application of physical restraints remain insufficient [10].

In nursing practice, nurses can make appropriate judgments and decisions regarding the application of physical restraints by considering the severity of the disease, treatment intensity, individual characteristics of the patient, work environment, ward atmosphere, and nursing staff size [11]. Nurses need a clear understanding of the correct application of physical restraints to reduce various issues and side effects that may occur in patients [12]. Changes in human behavior are assumed to occur through cognitive changes or restructuring. Additionally, according to rational emotional behavior therapy, cognition affects emotions and behavior, emotions affect thinking and behavior, and cognition plays a central role. If this is applied, awareness, knowledge, and attitude can be cited as the main factors affecting restraint-related nursing practice. Having accurate information and knowledge about the application of physical restraints is crucial for nurses as it can help transform their attitudes and enhance their nursing practices. Therefore, it is essential and urgent to assess the perception, attitude, and knowledge of nurses regarding the application of physical restraints to establish clear criteria based on knowledge and ethical judgment and to prepare nurses for their effective nursing practices.

The nursing practice related to the application of physical restraints can be influenced by individual nurses' values, competencies, clinical experiences, work unit, and environmental factors [10]. When examining the research trends related to physical restraints both domestically and internationally, studies have primarily focused on understanding the current usage of physical restraints among intensive care unit nurses [3], exploring the relationship between knowledge and attitudes towards physical restraints [13], and developing and evaluating the effectiveness of educational programs for the proper application of physical restraints [14]. These studies have mainly targeted nurses in intensive care unit or long-term care hospitals, and there has been a relatively insufficient amount of research on identifying the factors that influence nursing practice related to physical restraint application among nurses in general hospitals.

Thus, this study aimed to assess the level of perception, knowledge, attitude, and level of nursing practice regarding the use of physical restraints among nurses in general hospitals in South Korea. The objective was to provide basic data for establishing strategies as well as supporting data for effective application of physical restraints for nurses.

## Methods

### Research design

This was a descriptive and cross-sectional design whereby a survey was used to collect data.

### Participants

The participants of this study were a convenience sample of nurses working at general hospitals located in Jeonbuk, Korea. They voluntarily agreed to participate in the research after understanding its purpose. Nurses who had less than three months of experience were excluded from the study as they are in a period focused on acquiring job-related knowledge and skills. We performed a power analysis to determine the appropriate sample size using computer program G-power version 3.1.9. Based on a significance level (α) of 0.05, power of 0.95, moderate effect size (0.15), and 10 predictors, the calculation revealed that 172 participants were required for detection. Considering a dropout rate of approximately 10%, a total of 200 questionnaires were distributed, of which 190 were collected. Ten incomplete questionnaires were excluded, leaving a total of 180 questionnaires for final analysis.

### Measurement

#### General characteristics

The general characteristics of the nurses included in this study were assessed through seven questions related to gender, age, education level, religion, work unit, clinical experience, and education experiences on physical restraints.,

#### Perceptions of physical restraint use

Perceptions of the use of physical restraints represent a person's thoughts about how important they believe the use of physical restraints is. To assess the perceptions about the use of physical restraints, the PRUQ (Perceptions of Restraint Use Questionnaire) was utilized [15]. Two bilingual nursing majors translated the tool, which was developed in America and whose validity and reliability were not verified in Korea. As a result of comparing the two translations, no items with semantic differences were found. Expert validity was conducted to ensure that the contents and instructions of the translated questionnaire were clearly understood. The expert group was comprised of two professors majoring in fundamental nursing and one nurse who had worked at the hospital for more than 10 years, following Lynn's standard that the number of people should be less than 3–10. The content validity index(CVI) of each item was calculated using a 4-point likert scale. The CVI was found to be 0.92 on average, and all items were above 0.80. In addition, prior to the main survey, a preliminary survey was conducted on 20 nurses to complete the questionnaire. The PRUQ consists of 17 items that inquire about the reasons for using restraints in general. It uses a 5-point Likert scale, ranging from 17 to 85, where higher scores indicate a more positive perception of restraint use. The reliability of the original tool [15] was reported with a Cronbach's α of .94, and in this study, the reliability was determined to be Cronbach's α of .90.

#### Knowledge of physical restraint use

Knowledge about physical restraints refers to the extent to which individuals possess accurate information regarding the appropriate use of restraints when applied to patients in a given situation. To assess knowledge about the use of physical restraints, a modified and revised version [16] of the Physical Restraint Questionnaire [17] was used. Each item was answered with "Yes" or "No." Scores ranged from 0 to 19, with higher scores indicating a higher level of knowledge regarding the use of physical restraints. The reliability of the original tool [16] was reported with a Kuder–Richardson Formula 20 (KR-20) = .74, and in this study, the reliability coefficient was determined to be Kuder–Richardson Formula 20 (KR-20) = .73.

#### Attitude of physical restraint use

Attitude refers to one's stance or thoughts regarding the application of physical restraints to patients, representing a disposition towards a particular object or situation. Attitudes towards the use of physical restraints were measured using the 12 items from the "Attitudes Regarding Use of Restraints" section of the Physical Restraint Questionnaire [18]. Two bilingual nursing majors translated the tool, which was developed in America and whose validity and reliability were not verified in Korea. As a result of comparing the two translations, no items with semantic differences were found. Expert validity was conducted to ensure that the contents and instructions of the translated questionnaire were clearly understood. The expert group was comprised of two professors majoring in fundamental nursing and one nurse who had worked at the hospital for more than 10 years, following Lynn's standard that the number of people should be less than 3–10. The content validity index(CVI) of each item was calculated using a 4-point likert scale. The CVI of all items were above 0.80. In addition, a preliminary survey was conducted on 20 nurses prior to the main survey. This tool utilized a 3-point scale (1: Agree, 2: Neutral, 3: Disagree) for each item. In this study, the reliability was determined to be Cronbach's α of .83.

#### Nursing practice of physical restraint use

Nursing practice of physical restraint use refers to the ability of nurses to demonstrate appropriate knowledge, judgment, skills, and attitudes necessary for competent performance in physical restraints use. To measure nursing practice related to the use of physical restraints, a tool developed by Janelli et al. [17] and modified by Choi and Kim [14] was utilized. The tool consisted of 14 items rated on a 3-point scale (1: Not at all, 2: Sometimes, 3: Always), with higher scores indicating a higher level of competence in performing appropriate practices related to the use of physical restraints. The reliability of the original tool [14] was reported with a Cronbach's ⍺ of .73. In this study, the reliability was determined to be Cronbach's ⍺ of .79.

### Data collection and ethical approval

The data collection period was from October 1st to October 30th, 2022. Prior to data collection, the research obtained approval from the Institutional Review Board (IRB) of the researcher's affiliated institution (IRB No. 202209-SB-090). Researchers visited regular meetings for each ward. In the meeting room, participants completed the questionnaires, and the survey took approximately 15–20 min to complete. Before collecting data, the purpose and procedures of the study were explained to the participants, and a written informed consent form was obtained to protect the rights of the participants. To ensure honest responses, the consent form was printed separately for participants to sign, and the completed questionnaires were sealed in return envelopes for submission. All collected data were anonymized and coded, and it was explained that the data would be securely discarded at the end of the study. It was also emphasized that the collected data would not be used for any purpose other than the research objectives.

### Data analysis

The collected data were analyzed using SPSS WIN 28.0 software. Descriptive statistics were used to analyze the general characteristics of the participants. Perceptions, knowledge, attitudes, and nursing practice related to the use of physical restraints were calculated using means and standard deviations. Nursing practice related to the application of physical restraints according to general characteristics was analyzed using t-tests and ANOVA. The correlation between measured variables was examined using Pearson's correlation coefficient. Multiple linear regression analysis was used, and the dependent variable was nursing practice of patient physical restraint. The independent variables were selected as variables that showed significant differences in nursing practice of patient physical restraint among general characteristics, along with perceptions, knowledge, and attitude toward physical restraints.

Skewness and kurtosis were analyzed to check the normality of the data used in this study. The absolute value of skewness was 0.093–1.119, less than 2, and the absolute value of kurtosis was 0.125–0.975, less than 7, satisfying normality, so the parametric statistical method was applied.

## Results

### General characteristics and differences in nursing practice of physical restraint use according to the general characteristics

The total number of participants was 180, with females accounting for 88.3% of the sample. The majority of participants were below the age of 30, comprising 46.1% of the total. Regarding education level, 58.9% had a bachelor's degree, and 61.1% reported no religious affiliation. In terms of total clinical experience, 36.7% had less than 5 years. The most common department of employment among the participants was the intensive care unit (35.0%), and 71.1% of the participants reported having received education on the use of physical restraints (Table 1).

**Table 1:** Differences in nursing practice of physical restraint use according to the general characteristics to sociodemographic profile (*n* = 180)

Characteristics	Categories	n(%)	Mean ± SD	t/F	*p*	Scheffé
Gender	Male	21 (11.7)	2.57 ± 0.27	-1.24	.216	
	Female	159(88.3)	2.65 ± 0.26			
Age (year)	1 < 30	83 (46.1)	2.67 ± 0.24	1.68	.174	
	30–39	41 (22.8)	2.58 ± 0.31			
	40–49	43 (23.9)	2.61 ± 0.28			
	≥ 50	13 ( 7.2)	2.73 ± 0.19			
Education level	College degree	26 (14.4)	2.67 ± 0.23	0.39	.678	
	UBachelor	106(58.9)	2.63 ± 0.27			
	≥ Master	48 (26.7)	2.65 ± 0.26			
Religion	Yes	70 (38.9)	2.65 ± 0.25	0.42	.733	
	No	110(61.1)	2.63 ± 0.27			
Clinical experience (year)	< 5	66 (36.7)	2.63 ± 0.27	1.16	.325	
	5–9	44 (24.4)	2.62 ± 0.27			
	10–19	23 (12.8)	2.73 ± 0.18			
	≥ 20	47 (26.1)	2.62 ± 0.28			
Work unit	Medical ward^a^	23 (12.8)	2.57 ± 0.31	8.33	< .001	a,b,d < c
	Surgical ward^b^	52 (28.9)	2.57 ± 0.23			
	ICU^c^	63 (35.0)	2.77 ± 0.19			
	Emergency room^d^	32 (17.8)	2.51 ± 0.31			
	Outpatient department^e^	10 ( 5.5)	2.68 ± 0.18			
Education experiences on physical restraints	Yes	128 (71.1)	2.68 ± 0.25	3.53	< .001	
	No	52 (28.9)	2.53 ± 0.27			

*ICU* intensive care unit, including general intensive care unit, respiratory intensive care unit, neonatal intensive care unit and emergency intensive care unit

Nursing practice of physical restraint use showed statistically significant differences based on the work unit and education experience on the use of physical restraints. Post hoc analysis showed that nurses in the intensive care unit had higher scores in nursing practice related to the use of physical restraints compared to those in medical ward, surgical ward, and emergency room (Table 1).

### Degrees of mean scores perceptions, knowledge, attitude, and nursing practice related to physical restraints use

The participants' average score for perceptions related to the use of physical restraints was 3.66 ± 0.54 out of 5. Regarding attitudes, the average score was 1.73 ± 0.22 out of 3. Participants' knowledge related to the use of physical restraints had an average score of 12.08 ± 2.03 out of 19. In terms of nursing practice, the average score was 2.64 ± 0.26 out of 3 (Table 2).

**Table 2:** Perceptions, knowledge, attitude, and nursing practice related to physical restraints use (*n* = 180)

Variables	Mean ± SD	Min∼Max	Range
Perception	3.66 ± 0.54	2.47∼5.00	1∼5
Knowledge	12.08 ± 2.03	6.00∼16.00	0∼19
Attitude	1.73 ± 0.22	1.17∼2.58	1∼3
Nursing practice	2.64 ± 0.26	1.64∼3.00	1∼4

### Correlations among perceptions, knowledge, attitude, and nursing practice related to the physical restraints use

The analysis of correlations between perceptions, knowledge, attitudes, and nursing practice related to the use of physical restraints revealed that attitudes towards the use of physical restraints had a negative correlation with knowledge (*r* = -0.32, *p* < 0.001). Knowledge showed a positive correlation with nursing practice (*r* = 0.28, *p* < 0.001). The strength of the correlation between statistically significant variables is weak (Table 3).

**Table 3:** Correlations among perceptions, knowledge, attitude, and nursing practice related to physical restraints use (*n* = 180)

	Attitude r (*p*)	Knowledge r (*p*)	Nursing practice r (*p*)
Perception	.05 (.468)	.11 (.155)	.11 (.155)
Attitude	1	-.32 (< .001)	-.12 (.107)
Knowledge		1	.28 (< .001)
Nursing practice			1

### Factors affecting nursing practice related to the physical restraints use

To identify the factors affecting nursing practice related to the use of physical restraints, variables that showed significant differences in nursing practice among the general characteristics, such as work unit and education experience on the use of physical restraints, were included as independent variables along with perceptions, knowledge, and attitude. Work unit and education experience on the use of physical restraints were treated as dummy variables. The Variance Inflation Factor (VIF) was examined to assess multicollinearity among the independent variables, and the results ranged from 1.08 to 1.99, indicating no significant multicollinearity. Furthermore, the Durbin-Watson statistic of 1.950 indicated no auto correlation, indicating the suitability of the data for multiple regression analysis. The results of the analysis revealed that knowledge (β = .23, *p* = 0.002), work unit (intensive care unit) (β = .43, *p* < 0.001), and education experiences on physical restraints (Yes) (β = .18, *p* = 0.009) were statistically significant predictors of nursing practice. The regression model used in this study was statistically significant (*F* = 7.45, *p* < 0.01), and the overall explanatory power was 22% (Table 4).

**Table 4:** Factors affecting nursing practice related to the physical restraints use (*n* = 180)

Variable	B	SE	*β*	t	*p*
(Constant)	2.09	0.23		9.11	< .001
Perception	0.02	0.03	.05	0.71	.478
Attitude	-0.06	0.08	-.05	-0.67	.506
Knowledge	0.03	0.01	.23	3.18	.002
Work unit (Medical ward)^a^	0.07	0.06	.09	1.14	.257
Work unit (Surgical ward)^a^	0.08	0.05	.13	1.43	.154
Work unit (Intensive care unit)^a^	0.24	0.05	.43	4.65	< .001
Work unit (Outpatient department)^a^	0.09	0.09	.08	1.08	.281
Education experiences on physical restraints (Yes)^a^	0.11	0.04	.18	2.66	.009

*F* = 7.45, *R*^2^ = .26, Adj *R*^2^ = .22, *p* < .001

*SE* Standard error, *Adj* Adjusted, *B* unstandardized coefficient, *β* standardized coefficient

^a^Dummy variable: work unit (0 = Emergency Room), Education experience (0 = No)

## Discussion

This study aimed to analyze nurses' perceptions, knowledge, attitudes, and nursing practice related to the use of physical restraints in order to identify factors that have a significant impact on the actual application of physical restraints. The goal was to contribute foundational data for preventing inappropriate use of physical restraints and developing regulations and practical guidelines related to their use. The following implications can be drawn from the study results.

Among the study participants, 71.1% had received education on the use of physical restraints, indicating a high implementation rate of education on this topic. However, it was found that 72.2% of the participants did not recognize bedrails as a form of physical restraint, and 66.6% of the participants in a previous study perceived their education on the topic as inadequate, highlighting the need for proper education on the application of physical restraints [19].

The perception score regarding the use of physical restraints was 3.66 (out of 5), indicating an overall positive perception towards the use of physical restraints for physical confinement. This finding is consistent with previous studies [20] and suggests that nurses perceive the use of physical restraints as an important and valuable nursing intervention in specific situations. The factors that were considered important by the study participants regarding the application of physical restraints were ranked in the following order: "To prevent the removal of catheters," "To prevent the removal of nasogastric tubes," "To prevent the removal of intravenous injections," and "To protect the patient from falling out of bed." These findings align with similar results reported in previous studies [21, 22]. Therefore, it can be interpreted that nurses perceive the application of physical restraints as highly important in terms of protecting the patient's safety and preventing medical procedures from being compromised. On the other hand, the items such as "To substitute for the observation by the nursing provider," "To prevent the patient from taking other people's belongings," "To provide quiet time or rest for patients exhibiting excessive behaviors," and "To protect wandering patients" were considered less important in the application of physical restraints. This suggests that physical restraints are not applied for the convenience of nurses.

Despite the fact that the use of physical restraints is intended for patient safety, it can also lead to various physical and psychological damages [6, 7, 23]. In light of this, it is necessary to include education on the negative effects associated with physical restraint use when training nurses. Although there are currently various opportunities for human rights education to protect individuals' rights at the national level, the perception of healthcare providers regarding physical restraints still shows limited changes in clinical practice. Physically restraining patients is ultimately aimed at ensuring safer and more effective treatment and nursing care [24]. However, when alternative physical restraint methods developed with the inclusion of restraint guidelines were applied, there was no difference in the duration of physical restraint use and the use of alternative methods [25]. Therefore, there is a need for the development of educational programs that enable healthcare providers to prioritize appropriate alternatives over relying solely on physical restraints, and to promote a shift in perception regarding the use of physical restraints.

The level of knowledge regarding the use of physical restraints was 12.08 out of 19 points, which is similar to the results of a study conducted on nursing providers in elderly facilities using the same tool [16]. The high overall knowledge level can be attributed to the fact that the majority of the participants had received education on physical restraints. Unsafe patient behavior can lead to the application of physical restraints. Nurses should be able to identify the psychological, physical, and environmental causes of problematic behavior in patients and be competent in managing them. Additionally, having the correct knowledge is crucial for determining the appropriate application sites for restraints and deciding when to initiate or discontinue their use. Therefore, improving evidence-based knowledge related to restraint application is important for reducing the use of physical restraints. It is necessary to develop educational programs aimed at reducing the use of physical restraints based on previous studies that have shown an improvement in overall knowledge levels through systematic and ongoing education [14, 26].

When examining the answer rates for the knowledge items, the item "A record should be kept on every shift of patients in restraints." had the highest correct answer rate of 96.1%. This indicates that nurses are well aware of the importance of documentation and its association with legal responsibility. The item " Restraints are a device used to prevent injuries." had a correct answer rate of 95%, which aligns with the findings [27] emphasizing the importance of protecting patients from falls and injuries. Raising bed rails as a priority intervention to prevent falls can be considered a form of restraint. On the other hand, the item "A patient should never be restrained while lying flat in bed because of the danger of choking" had the lowest correct answer rate of 11.1%. This reflects a lack of awareness regarding the potential risk of suffocation-related deaths due to restraints. Particularly, the correct answer rate for this item differs significantly from the findings [18] with a 57% correct answer rate and Suen et al. with a 40% correct answer rate [28]. In Korea, wrist and limb restraints are predominantly used [5], indicating a lack of knowledge regarding chest restraints. The item "When a patient is restrained in a bed, the restraint should not be attached to the side rails." had a correct answer rate of 48.9%, which was higher than the 32.9% correct answer rate in the study [16], but significantly lower than the 98% correct answer rate reported [18]. This suggests that restraints are frequently applied to bedrails in Korea, despite the absence of specific guidelines or regulations on the application of restraints.

The overall knowledge score of the study participants was moderate, but there were several items with low correct answer rates, indicating a limited and fragmented knowledge of physical restraints. Despite this, frequent use of restraints in patient care suggests the need to improve the level of knowledge regarding restraint use. Considering the challenges of nurses' shift work, the development and implementation of web-based educational programs or other forms of education that are not time-constrained could enhance the effectiveness of education on restraint use [14]. Therefore, there is a need for various forms of education in the clinical setting to provide nurses with specialized knowledge on restraint use.

The attitude towards restraint use was moderate, with a score of 1.73 (out of 3). The items with the highest scores were "The hospital is legally responsible to use restraints to keep the patient safe." and "I feel embarrassed when the family enters the room of a patient who is restrained and they have not been notified." These results indicate a strong belief in the appropriate use of restraints, emphasizing that restraints should be used for the benefit of the patient rather than causing harm. It also reflects nurses' awareness of their responsibility in restraint use and their ethical attitude valuing the rights of patients and their families. The attitude measurement items regarding restraint use include topics such as patient, family, and healthcare professionals' right to refuse restraints, facility and staffing considerations, emotional aspects of restraint application, ethical conflicts, decision-making processes, and regulations and guidelines related to restraints. Continuous interventions addressing these topics are necessary to promote the development of appropriate attitudes towards restraint use in nursing practice.

The formation of meaningful attitudes among nurses regarding the use of restraints can be accompanied by an ethical process in which nurses carefully consider the appropriateness of restraints as a therapeutic tool from the perspective of the patient as a whole. Therefore, it is necessary to provide ethical education that can reduce nurses' ethical dilemmas when applying restraints in real clinical settings. Through this, nurses can establish correct and positive beliefs, enabling them to provide efficient restraint nursing interventions that consider the patient's perspective.

The score for nurses' restraint nursing practice related to the use of physical restraints was high, with a mean of 2.64 out of 3. This finding is consistent with a previous study that examined the restraint nursing practice of nurses in medical-surgical units using the same tool [29]. Recent healthcare facility assessments indicate that nurses have a good understanding of the regulations and guidelines regarding the use of physical restraints and how to use them and document them in practice. However, while we can assess the nurses' level of understanding, it is necessary to directly observe whether they are implementing their understanding in practice. In addition, continuous education should be conducted in parallel so that desirable nursing practice can be achieved based on previous studies [14, 19] in which nursing performance scores have increased through the provision of education to reduce the use of restraints.

When analyzing the nursing practice related to the use of physical restraints on a item-by-item basis, the items "Explaining the reasons for applying restraints to family members" and "Explaining the reasons for applying restraints to patients" scored the highest. This is consistent with the findings of a previous study that also reported high scores for these items [30]. The research participants demonstrated a good understanding of patient rights in clinical settings and adequately explained the reasons for using restraints to patients and their families. On the other hand, the item with the lowest score was "Using restraints on more patients when there are fewer healthcare providers than when there are more healthcare providers," which is also consistent with the findings [29]. This result suggests that the use of physical restraints may vary depending on the number and ratio of patients cared for by healthcare providers. Additionally, the item "Most of the staff in our hospital prefer finding ways to control patient behavior rather than using physical restraints" received a low score, indicating a lack of awareness regarding various alternative methods and advantages that should be considered before applying restraints. In future education programs, it is necessary to comprehensively address the topic of restraints, focusing not only on nursing actions but also on evidence-based practice and proactive exploration of restraint alternatives. By identifying the root causes of problematic behavior before applying restraints and understanding the various types and advantages of restraint alternatives, the unnecessary use of restraints can be reduced. The analysis of nursing practice related to the physical restraints use according to general characteristics revealed significant differences based on the work unit and education experience on the use of physical restraints. Nurses in the intensive care unit had higher scores in nursing practice related to the use of physical restraints compared to those in medical ward, surgical ward, and emergency room. In clinical practice, it is believed that creating a ward-specific protocol for the use of restraints can help nursing work, and it is necessary to develop an education program related to restraints and provide information on the use of restraints through continuing education.

In this study, an examination of the relationship between knowledge, attitudes, and nursing practice regarding physical restraints revealed that higher knowledge scores were associated with more negative and avoidance attitudes towards restraint use. Furthermore, a stronger negative and avoidance attitude was associated with higher nursing practice. These findings are consistent with previous studies that reported a negative attitude towards restraint use when knowledge about restraints was high [30, 31]. Although perception and nursing practice did not show significant correlations in this study, a positive correlation was observed. It is believed that the nursing practice was overrated by self-assessment surveys, as the surveys evaluated nursing practice based on the nurses' ethical awareness rather than actual practice. A study on nurses' understanding of ethical dilemmas related to restraint application [32] suggested that ethical awareness could influence positive nursing practice. Therefore, it is possible that the higher ratings of nursing practice were influenced by ethical consciousness. It is recommended that future research includes direct observation or objective assessments of nursing practice, as well as studies examining the relationship between ethical attitudes and nursing practice.

This study identified knowledge, work unit (intensive care unit), and education experience (yes) as factors influencing nursing practice in relation to physical restraint use. Specifically, work unit and education experience on physical restraints were found to be significant factors influencing nursing practice and highlighted the importance of acquiring accurate evidence-based knowledge about the physical restraints used in clinical practice. As nurses' evidence-based knowledge about restraint use increases, their attitudes towards restraint application become more negative, ultimately resulting in a decrease in the use of physical restraints in nursing practice [33, 34]. Based on the findings of this study, it is crucial to provide various education programs aimed at enhancing evidence-based knowledge to nurses. As part of the education, efforts should be made to transform the concept of bed rails from a means of fall prevention and patient safety to that of physical restraints, following the international definition of physical restraints [35]. In this study, only 27.8% of the participants considered bed rails as physical restraints for fall prevention and patient safety, indicating the need for ongoing awareness and further research on how bed rails are used as physical restraints. The work unit was also identified as a significant influencing factor, highlighting the need for future studies to compare nursing practice in restraint application across different work unit. Based on the results of this study, it is necessary to establish systematic guidelines for the application of physical restraints, taking into account variations in work unit and assigned responsibilities.

Although there is a limitation that the degree of correlation coefficient is low, this study has significance in empirically examining the relationship between nurses' perception, knowledge, attitudes, and nursing practice regarding the use of physical restraints. Based on the findings of this study, at the individual level, nurses should consider participating in ethical education and job-related training related to physical restraints. At the organizational level, clear guidelines and regulations regarding the use of physical restraints need to be established. At the national level, there is a need to establish legal grounds that enable nurses to enhance their ethical awareness in the application of physical restraints.

One limitation of this study is that the reliability of the knowledge instrument regarding the use of physical restraints [17] was somewhat low. This tool was developed in a foreign country in 1991 and is still in use, but there is a need for the development and validation of new tools that are applicable to the current environment, considering the changing circumstances. Additionally, the assessment of nursing practice was based on self-report surveys, which could have resulted in overestimation or underestimation of actual performance. Therefore, there is a need for assessments based on direct observation to ensure accurate evaluation. Because the subjects of this study were a convenience sample of nurses from one geographical location in South Korea, the results of this study cannot be generalized to other nurses working in other hospitals. Therefore, there is a need for repeated research with different subjects, regions, and hospital sizes.

## Conclusions

This study is significant in that knowledge of physical restraints, educational experience, and work units are the strongest predictors of nursing practice for patients' use of physical restraints. The findings of this study can serve as foundational data for the development of evidence-based educational programs on restraint application targeting nurses. Ultimately, it can improve nurses' knowledge and encourage positive attitudes about the use of physical restraints, minimizing potential risks and concerns.

## Data Availability

The datasets during and / or analyzed during the current study available from the corresponding author on reasonable request.

## References

[1] Rose L, Dale C, Smith OM, Burry L, Enright G, Fergusson D, Sinha S, Wiesenfeld L, Sinuff T, Mehta S (2016). A mixed-methods systematic review protocol to examine the use of physical restraint with critically ill adults and strategies for minimizing their use. Syst Rev.

[2] Enns E, Rhemtulla R, Ewa V, Fruetel K, Holroyd-Leduc JM (2014). A controlled quality improvement trial to reduce the use of physical restraints in older hospitalized adults. J Am Geriatr Soc.

[3] Hevener S, Rickabaugh B, Marsh T (2016). Using a decision wheel to reduce use of restraints in a medical-surgical intensive care unit. Am J Crit Car.

[4] Minnick AF, Mion LC, Johnson ME, Catrambone C, Leipzig R (2007). Prevalence and variation of physical restraint use in acute care settings in the US. J Nurs Scholarsh.

[5] Kim MY, Park JS (2010). A study on the application of physical restraints in intensive care units. J Korean Acad Fundam Nurs.

[6] Kandeel NA, Attia AK (2013). Physical restraints practice in adult intensive care units in Egypt. Nurs Health Sci.

[7] Berzlanovich AM, Schöpfer J, Keil W (2012). Deaths due to physical restraint. Dtsch Arztebl Int.

[8] Joint Commission on Accreditation of Healthcare Organization (2006). Comprehensive accreditation manual for behavioral healthcare.

[9] Korea Institute for Healthcare Accreditation (2020). Hospital certification criteria on 2015.

[10] Kang KJ, Kim EM, Ryu SA (2011). Factors influencing clinical competence for general hospital nurses. J Korea Contents Assoc.

[11] Ko YJ, Ha SM (2019). Physical restraints use and associated factors among elderly patients in long-term care hospitals. JKAIS.

[12] Kim EM, Park YK, Suh SR (2018). Knowledge, attitude, perception, and nursing skills about physical restraints among nursing personnel in long-term care hospitals according to physical restraints guideline. AJMAHS.

[13] Eskandari F, Abdullah KL, Zainal NZ, Wong LP (2017). Use of physical restraint: nurses’ knowledge, attitude, intention and practice and influencing factors. J Clin Nurs.

[14] Choi K, Kim J (2009). Effects of an educational program for the reduction of physical restraint use by caregivers in geriatric hospitals. J Korean Acad Nurs.

[15] Evans LK, Strumpf NE, Perry HM, Morley JE, Coe RM (1993). Frailty and physical restraint. Aging and musculoskeletal disorders.

[16] Kim SM, Lee YJ, Kim DH, Kim SY, Ahn HY, Yu SJ (2009). Perception, attitude, and knowledge about physical restraints among nursing personnels in long term care facilities. J Korean Acad Soc Nurs Educ.

[17] Janelli LM, Scherer YK, Kanski GW, Neary MA (1991). What nursing staff members really know about physical restraints. Rehabil Nurs.

[18] Janelli LM, Stamps D, Delles L (2006). Physical restraint use: a nursing perspective. Medsurg Nurs.

[19] Fariña-López E, Estévez-Guerra GJ, Gandoy-Crego M, Polo-Luque LM, Gómez-Cantorna C, Capezuti EA (2014). Perception of Spanish nursing staff on the use of physical restraints. J Nurs Scholarsh.

[20] Myer H, Nikoletti S, Hill A (2001). Nurses’ use of restraints and their attitudes toward restraint use and the elderly in an acute care setting. Nurs Health Sci.

[21] Kim JS, Oh HY (2006). Perceptions and attitude on use of physical restraints among caregivers in long term care facilities. J Korea Gerontological Soc.

[22] Hamers PH (2015). Review: nurses predominantly have negative feelings towards the use of physical restraints in geriatric care, though some still perceive a need in clinical practice. Evid Based Nurs.

[23] Evans D, Wood J, Lambert L (2003). Patient injury and physical restraint devices: a systematic review. J Adv Nurs.

[24] Kim KS, Kim JH, Lee SH, Cha HK, Shin SJ, Chi SA (2000). The physical restraint use in hospital nursing situation. J Korean Acad Nurs.

[25] Wang L, Zhu XP, Zeng XT, Xiong P (2018). Nurses’ knowledge, attitudes and practices related to physical restraint: a cross-sectional study. Int Nurs Rev.

[26] Kong EH (2012). Development and evaluation of a web-based education program to reduce restraint use for nursing home caregivers. J Korean Gerontol Nurs.

[27] Leahy-Warren P, Varghese V, Day MR, Curtin M (2018). Physical restraint: perceptions of nurse managers, registered nurses and healthcare assistants. Int Nurs Rev.

[28] Suen LK, Lai CK, Wong TK, Chow SK, Kong SK, Ho JY, Kong TK, Leung JS, Wong IY (2006). Use of physical restraints in rehabilitation settings: staff knowledge, attitudes and predictors. J Adv Nurs.

[29] Hong JE. Nurse’s Knowledge, Attitudes and nursing practice on physical restraints in critical care unit. Seoul: Unpublished doctoral dissertation, Yonsei University; 2018.

[30] Yeo JM, Park MH (2006). Effects of education program for nurses on the use of restraints. J Korean Acad Nurs.

[31] Ha SM (2019). Factors influencing nursing practices of physical restraint use among nurses working in long-term care hospitals. JKAIS.

[32] Ye JR (2018). Physical restraints: an ethical dilemma in mental health services in China. Int J Nurs Sci.

[33] Luk E, Burry L, Rezaie S, Mehta S, Rose L (2015). Critical care nurses’ decisions regarding physical restraints in two Canadian ICUs: a prospective observational study. Can J Crit Care Nurs.

[34] Suliman M, Aloush S, Al-Awamreh K (2017). Knowledge, attitude and practice of intensive care unit nurses about physical restraint. Nurs Crit Care.

[35] Bleijlevens M, Wagner L, Capezuti E, Hamers J (2016). Physical restraints: consensus of a research definition using a modified delphi technique. J Am Geriatr Soc.

